# Gut–Brain Axis: Role of Gut Microbiota on Neurological Disorders and How Probiotics/Prebiotics Beneficially Modulate Microbial and Immune Pathways to Improve Brain Functions

**DOI:** 10.3390/ijms21207551

**Published:** 2020-10-13

**Authors:** Kanmani Suganya, Byung-Soo Koo

**Affiliations:** 1 Department of Oriental Medicine, Dongguk University, Gyeongju 38066, Korea; suganyawinning2007@gmail.com; 2 Department of Oriental Neuropsychiatry, Graduate School of Oriental Medicine, Dongguk University, Ilsan Hospital, 814 Siksa-dong, Goyang-si, Gyeonggi-do 10326, Korea

**Keywords:** gut microbiota, gut–brain axis, neurological disorders, probiotics, antibiotics

## Abstract

The gut microbiome acts as an integral part of the gastrointestinal tract (GIT) that has the largest and vulnerable surface with desirable features to observe foods, nutrients, and environmental factors, as well as to differentiate commensals, invading pathogens, and others. It is well-known that the gut has a strong connection with the central nervous system (CNS) in the context of health and disease. A healthy gut with diverse microbes is vital for normal brain functions and emotional behaviors. In addition, the CNS controls most aspects of the GI physiology. The molecular interaction between the gut/microbiome and CNS is complex and bidirectional, ensuring the maintenance of gut homeostasis and proper digestion. Besides this, several mechanisms have been proposed, including endocrine, neuronal, toll-like receptor, and metabolites-dependent pathways. Changes in the bidirectional relationship between the GIT and CNS are linked with the pathogenesis of gastrointestinal and neurological disorders; therefore, the microbiota/gut-and-brain axis is an emerging and widely accepted concept. In this review, we summarize the recent findings supporting the role of the gut microbiota and immune system on the maintenance of brain functions and the development of neurological disorders. In addition, we highlight the recent advances in improving of neurological diseases by probiotics/prebiotics/synbiotics and fecal microbiota transplantation via the concept of the gut–brain axis.

## 1. Introduction

In the human body, trillions of microbes reside that are assumed to influence and regulate the physiology of the host. Most microbes are inhabitants in the gastrointestinal tract of the humans and are known as gut microbiota (GM). Several factors affect the inhabitants and the composition of the GM. Therefore, it can be varied depending on the host nature. The GM is comprised of four major (*Bacteroidetes*, *Firmicutes*, *Proteobacteria*, and *Actinobacteria)* and two minor phyla (*Verrucomicrobia* and *Fusobacteria*) [1]. These commensal bacteria not only communicate with each other but also with host gut epithelium, to maintain the gut homeostasis and improve the host immunity. The gut-residing microbes exhibit several beneficial effects on the host at healthy state, but in the disease or disruption state, it has been reported to be involved in the progression of several diseases, including neurological disorders [2]. Growing evidence suggests that the gut has strong bidirectional communication with the brain, which is vital for maintaining the brain functions and gut homeostasis. Neurological disorders, such as Parkinson’s disease (PD), Alzheimer’s disease (AD), multiple sclerosis (MS), autism spectrum disorder (ASD), and stress are believed to induce changes in the bidirectional relationship, which results in the induction of brain–gut disorders, such as irritable bowel syndrome (IBS) and others [3,4]. In addition, the dietary pattern, antibiotic usage, and bacterial and viral infections are often associated with the alternation of gut bacterial composition and the loss of gut homeostasis, which have been implicated in the pathogenesis of gut–brain disorders [5]. However, there is still remarkable controversy on the molecular mechanism behind the alternation of the gut–brain bidirectional relationship. Germ-free (GF) mice exhibit abnormalities in the development of the gastrointestinal tract, compared to specific pathogen-free (SPF) and GF-conventional mice, suggesting that commensal gut bacteria are important for the early postnatal development of the enteric nervous system (ENS) in the intestine [6,7]. Similarly, McVey et al. [8] also reported that GM restores the normal intrinsic and extrinsic nerve function and gut–brain signaling in GF mice. These studies confirm the critical role of the GM and their impacts on the developmental process of the central nervous system (CNS) shortly after birth. Therefore, this review provides an overview of the current evidence that points to the involvement of gut microbiota and their metabolites and cellular components on the progression of several neurological diseases. Moreover, the various therapeutic interventions used to ameliorate these disorders are highlighted.

## 2. Interaction of Gut and Nervous System: Gut–Brain Axis 

**Figure 1:**
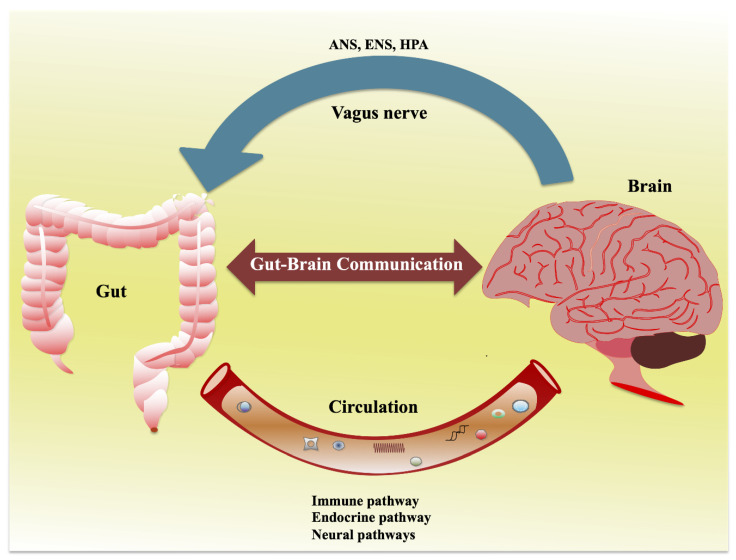
Schematic diagram showing the communication between the gut and brain. This is a bidirectional relationship that is strongly influenced by multiple pathways, including the autonomic nervous system (ANS), enteric nervous system (ENS), hypothalamic–pituitary–adrenal (HPA), immune pathways, endocrine pathways, and neural pathways.

Anatomically, the gut has a complex and bidirectional relationship with the CNS, which is termed as the gut–brain axis that crosstalk each other in the context of both health and diseases [3]. This crosstalk allows the gut sensory visceral signals that travel through the vagus nerve to influence the CNS to regulate the reflex and mind/moods changes; in turn, the brain directs the signals to modulate the gut physiology and other functions (Figure 1). Afferent (A neuron that brings signals in) and efferent (A neuron that carries signals out) neurons are involved in the connecting neuronal pathways and transferring the signals through different pathways, including the autonomic nervous system (ANS), enteric nervous system (ENS), hypothalamic–pituitary–adrenal (HPA) axis, sympatho-adrenal axis, and descending monoaminergic pathways [3,9]. Each system/pathway is intimately intertwined and regulated by several inter-relational and neurohumoral factors. The ENS is a complex network of neurons that is largely responsible for the intrinsic innervation of gut functions. It consists of two ganglionated plexuses, the myenteric and submucosal plexus, which regulate the gut functions, such as the gut motility (peristalsis), and secretion, and absorption [10]. 

The ENS communicates CNS through intestinofugal neurons to the ganglia of the sympathetic nervous system (SNS) with sensory information travel via primary afferent neurons follow vagal afferent routes in gut–brain signaling [10]. ANS is a neural relay network that is structured of sympathetic (Splanchic) and parasympathetic (vagal–sacral) nerves [3]. In association with neuronal and neuroendocrine signaling, ANS controls breathing, heartbeat, and CNS-mediated changes in the gut and its functions, such as digestion, gut motility and permeability, secretion of bile, carbohydrate levels, mechanical distortion of the mucosa, maintenance of epithelial fluid level, luminal osmolality, and production of mucus and mucosal immune response [3,11]. The ANS imparts direct neurological responses to the gut via CNS, resulting in changes of the gut physiology. The gut microbiota communicates with each other through their metabolites that sensed by the host cells and thereby interact with the ANS gut synapses [12]. Moreover, the ANS can affect the mechanisms of the gut epithelium that participates in the activation of the immune system directly by modification of the response of gut immune cells to microbes or indirectly via alternation of microbes to gut immune cells [13]. 

### 2.1. Gut–Brain Axis and the Microbiota

Growing evidence reports that gut microbiota has been shown to play a preeminent role in the gut–brain axis. Therefore, the interaction between the microbiota and brain is often called as microbiota/gut–brain axis [3]. This is a bidirectional relationship. Therefore, the disturbance in the gut bacterial composition might affect the neurological functions and vice versa [14]. Several studies suggest that the gut microbiota influence the development, functions and disorders of the CNS and ENS via the interaction and activation of pattern recognition receptors (PRRs) such as Toll-like receptors 2 and 4 (TLR2 and TLR4) [9,15]. Gut dysbiosis and associated loss of gut barrier integrity and intestinal permeability allow increasing translocation of the gut-bacteria-derived metabolites and microbes-associated molecular patterns (MAMPs) into mesenteric lymphoid tissues, resulting in the progression and development of various neurological diseases [16,17]. An animal study also reported that changes in the gut microbiota composition or lack of enteric bacterial composition in mice impaired lower in number of myenteric neurons and higher in incidence of bowel motor dysfunctions, which indicate the determinable effect of enteric bacteria on ENS tropism [6]. The GF animals also exhibited dysregulated hormone signaling, less expression of brain-derived neurotrophic factor (BDNF), differences in neurotransmission, and amino acid metabolism, as compared to conventional control mice [18,19,20]. Another study performed in Drosophila reported that gut microbes modulated the locomotor activity by increasing the production of metabolites [21].

An increased blood–brain barrier (BBB) and decreased occludin and claudin-5 expressions were observed in the frontal cortex, striatum, and hippocampus of GF mice [22]. *B. thetaiotaomicron* and *C. tyrobutyricum* colonization reduced the paracellular permeability by upregulating the expression levels of tight-junction proteins in GF mice [22]. In addition, remarkable abnormalities were observed in the ENS of GF mice, but these abnormalities were not apparent after the GF mice colonized with altered Schaedler flora (ASF), indicating the role of the specific bacterial flora on the development of ENS [7]. The GF mice also exhibited deficits in the intrinsic sensory signaling that is important for the CNS communication, could be restored in GF mice colonized with microbiota from specific pathogen-free (SPF) donor mice [8]. Moreover, changes in the antibiotic-mediated gut microbiota composition affect the structure and functions of ENS, neurochemistry, and decrease the number of ganglia residing enteric glial cells in vivo [23]. Evidence that in GF adult mice, the specific role of indigenous gut microbes on regulation of network of mucosal enteric glial cells migration and homeostasis, but that was not limited to the time of postnatal development [24]. Furthermore, infection in a mice model was used to access the pathogenic bacterial effects on the nervous system that mediates through the activation of host immune system. The mice with infected *Campylobacter rodentium* induced anxiety-like behavior via vagal sensory neurons; however, there were no differences in the plasma levels of proinflammatory cytokines compared to the control [25]. *Campylobacter jejuni* infection activates c-Fos, a neuronal activation marker in the vagal sensory ganglia and nucleus tractus solitaries (NTS) in the medulla oblongata of mice [26]. Based on these studies, the bidirectional relationship between gut and brain is a fundamental aspect that explains the synergistic association of the gut microbiota with host in accessing the signaling of the gut–brain axis to regulate the host behavior and emotional or moods/minds [27,28]. 

### 2.2. Microbial Metabolites and Cellular Components on CNS and ENS

**Figure 2:**
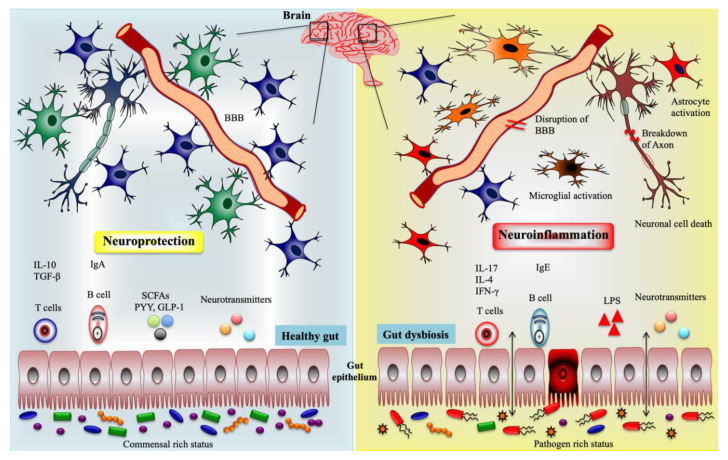
Influence of immune and metabolic regulation by the gut microbiota during the healthy and gut dysbiosis state via gut–brain axis. (Left side) The gut microbiota promote the production of short-chain fatty acids (SCFAs), gut-derived peptides, neurotransmitters, and regulatory T and B cells through interactions with intestinal immune cells. Along with this, the microbiota maintain intestinal permeability, decrease the production and translocation of lipopolysaccharides (LPS) to the periphery, reduce the blood–brain barrier BBB disruption, activate brain immune and neuronal cells, and improve brain functions. (Right side) Gut microbiota exhibit the opposite role to induce inflammatory state during the dysbiosis condition. Microbiota increase inflammatory T and B cells, LPS, and proinflammatory cytokines production and reduce the SCFAs level, thereby inducing the BBB permeability, activation of microglial cells and astrocytes, and neuroinflammation. Peptide YY (PYY), glucagon-like peptide-1 (GLP-1), and transforming growth factor-β (TGF-β).

Current studies have provided molecular insights into how the gut microbiota influences the CNS and ENS functions, implicating the vital role of gut microbiota-derived metabolites and cellular components on improvement of brain homeostasis, as well as the progression of neuropsychological problems. The gut microbiota could communicate CNS/ENS through the production of several metabolites/neurotransmitters with neuromodulatory properties. Among them, tryptophan precursors and metabolites, 5-hydroxytryptamine (5-HT), gamma-aminobutyric acid (GABA), glutamine, histamine, branched-chain amino acid (BCAAs), LPS, SCFAs, bile acids, and catecholamines are the important host/microbes-derived metabolites or components that modulates important processes that ensue during neurogenesis, glial cell function, myelination, synaptic pruning, and blood–brain barrier function [9,29] (Figure 2). 

Perturbation to the gut microbiota composition under these conditions could affect the development of CNS and ENS [9]. GABA is the prime inhibitory neurotransmitter in the host nervous system that has been produced by host/microbes through the conversion of amino acid glutamate [30]. Some probiotic strains have been shown to produce GABA in vitro and in vivo. Specifically, *Escherichia* spp. and *Lactobacillus* spp. were able to synthesize GABA [31,32]. The human intestinal isolates such *Lactobacillus* and *Bifidobacterium* increased the level of GABA in ENS [33], and its receptors largely distributed through ENS [34]. In an animal study, the administration of *L. rhamnosus* [JB-1] could modulate the mRNA level of GABA receptors that impair anxiety-like symptoms in mice via vagus nerve [35]; however, no clear mechanisms were found for GABA on gut–brain crosstalk. Evidence suggests that glutamate is also a neurotransmitter in the CNS/ENS. The modulation of glutamatergic receptors in the bidirectional relationship influence physiological responses in both gut and brain [36]. It can be produced by bacteria and from dietary source. Bacterial strains such as *Corynebacterium glutamicum* [37], *Brevibacterium* spp. [38], *L. plantarum*, and *L. lactis* [39], have been shown to produce glutamate that is absorbed by colonocytes and transferred the amino acid from the lumen to portal circulation [40]. Glutamine can also be synthesized by neurons in CNS, from α-ketoglutarate that originates from the tricarboxylic acid cycle, as well as from the glutamine deamination by glutaminase [41]. The glutamine was rich in the astrocytic cytosol that is transported into the extracellular fluid, where it is uptaken by neurons and converted it into glutamate by deaminase [42] and plays a role in the pathophysiological changes of neuronal excitability [43]. Similar to CNS, myenteric neurons may produce glutamate through the hydrolysis of glutamine [44], whereas the immunohistochemical co-localization of glutamate and glutamine was found in the nerve bundles and muscle layers of the rat stomach [45]. In addition, the presence of glutamate in the primary afferent neurons indicates that it may act as a sensory transmitter in the enteric neurons and transmitting the signals from the mucosal layer to ENS [46]. The increasing glutamatergic neurotoxicity is mainly through the differential activation of synaptic versus extrasynaptic N-methyl-D-asparate (NMDA) receptors [47,48]. It is clear that glutamate functions as a neurotransmitter in the gut–brain crosstalk.

Hydroxytryptamine (5-HT) is a neurotransmitter that can be produced by the gut microbiota from tryptophan with the help of enzyme tryptophan hydroxylase 1 (TPH1) [49]. The TPH-1 defective GF mice with gut microbes exhibited a lower number of enteric neurons than GF and conventional THP1 transgenic mice. Moreover, GF mice that received microbes from the conventional mice could modify the anatomy of ENS and intestinal transit rates that are associated with increasing production of neuronal and mucosal 5-HT production and the proliferation of enteric neuronal progenitors [49]. The presence of 5-HT receptor antagonist was differently affected the functions of the ENS, while treatment of GF mice with 5-HT receptors maintained the normal gut physiology, suggesting that the microbiota affect the ENS in a 5-HT dependent manner with intestinal transit rates changes in vivo [46]. TPH2 is also a rate-limiting enzyme in the production of 5-HT. TPH2-mutated mice showed abnormal ENS development, ENS-mediated gut functions than control mice [50]. Moreover, a decrease in level of 5-HT in enteric neurons, gut transit rates and colorectal motility were observed in mice mutated with THP2 that administrated with 5-HTP in slow release (5-HTP-SR) restored 5-HT enteric neurons, total intestinal transit and colonic motility, indicating that the production of 5-HT by neurons associates with depression [50]. *C. perfringens*, a member of the human and rodent gut microbiota, has been shown to increase colonic and blood 5-HT production via TPH1 in mice [51]. Moreover, the colonic 5-HT production is influenced not only by the gut microbes, but also by its metabolites (SCFAs) in vivo [51,52].

Short-chain fatty acids (SCFAs) are the important gut microbial metabolites produced from the fermentation of dietary fibers in the intestine tract. Acetate, propionate and butyrate are the principal SCFAs produced mainly by the *Bacteroidetes* and *Firmicutes* [53,54,55]. After production, these were absorbed immediately into the portal circulation and transferred to peripheral tissues including brain, where they play a crucial role in the regulation of neurological functions. SCFAs mediate the signals principally via interactions with orphan G protein-coupled receptors (GRP41 and GRP43, also known as free fatty acid receptors or FFAR3 and FFAR2, and GRP109A), Olfactory receptor 78 (Olfr78) and inhibition of histone deacetylases or HDACs [53,56,57]. Germ-free mice treated with SCFAs restored changes of microglial cell structure and also reversed immaturity of the cells [58]. SACFs potentially influence or affect the brain neurological functions via four important ways, including immune, vagal, endocrine and humoral pathways. SCFAs and their functions in the intestine are well addressed, and these also affect peripheral immune system to regulate brain functions. SCFAs directly induce immune cells including regulatory T cells, endocrine cells, and vagal neuronal cells to produce higher level of regulatory cytokines and gut-derived peptides to influence or modulate brain functions. SCFAs treatment improved the neurochemical phenotype of ENS and increased proportion of choline acetyltransferase (ChAT)-immuno reactive myenteric neurons in rat [59]. In addition, the silencing of monocarboxylate transporter 2 (Important for enteric neuron production) could prevent the butyrate induced ChAT (ChAT)-immunoreactive myenteric neurons in vivo [59], indicating that SCFAs might play role in the ENS homeostasis and physiology. Mice administrated with SCFAs alleviated psychosocial stress induced alternations, and increased responsiveness to stress and gut permeability in mice [60]. Moreover, SCFAs treatment could not affect the SCFAs receptors and FFA receptors 2/3, body weight gain, fecal SCFAs, colonic gene expression, and microbiota composition in mice, indicating the role of SCFAs on alleviation of psychosocial stress induced disorders in vivo [60]. In human and animal studies, a decrease in the level of fecal and intestinal SCFAs was observed with PD [61] and ALD [62]. Acute oral butyrate administration suppressed the activity of orexigenic neurons in the hypothalamus and the neuronal activity in the nucleus tractus solitaries (NTS) and dorsal vagal complex of mice, suggesting a dynamic regulation of butyrate on the gut–brain neuronal circuitry [63]. Stimulation of the rat microglial and hippocampal cells with butyrate reduced the LPS induced inflammatory response in vitro, but in murine microglial cells, it exhibits proinflammatory effects [64].

**Figure 3:**
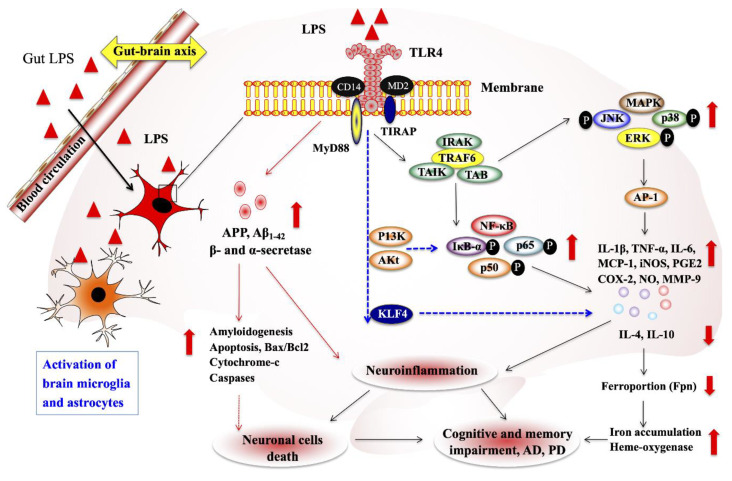
Gut–brain axis exacerbates neurological disorders through gut-microbiota-derived molecular patterns. The dysregulated LPS productions were able to translocate to the brain through the circulation, where it mediates various dangerous signaling by interacting Toll-like receptor 4 (TLR4), resulting in the induction of neuroinflammation and other neurological diseases. Lipopolysaccharide (LPS), Krüppel-like factor 4 (KLF4), nitric oxide (NO), prostaglandin E2 (PE2), matrix metalloproteinase-9 (MMP-9), and amyloid precursor protein (APP). (↑ ↓ indicate up and down-regulation).

Like metabolites, the gut microbiota-derived cellular components also affected the functions of CNS and ENS through the activation of TLRs expressed in enteric neurons, sensory afferent neurons and other cells of the brain [65,66]. LPS is a prime cell wall component of the gut bacteria that induces neuroinflammation and neurodegenerative disease via the activation of TLR in brain cells. Microglial cells and astrocytes are the prominent cell types that can express TLRs, especially TLR4. The presence of LPS activates TLR4 in both cell types to induce an inflammatory response in vitro and in vivo. It has been reported that LPS/TLR4 signaling on microglial cells directly or indirectly influence the CNS by increasing the expression of inflammatory cytokines in the CNS or gut [67]. In addition, it affects the neuronal survival in ENS [68]. Immature microglia were found in GF mice that exhibit lower response to LPS as compared with microglia from conventional mice [58]. The lipid A and core LPS signals, and co-localization of LPS with CD14/TLR4 receptors and lipoprotein receptors (SR-BI, APoER2, and LDLr) were observed in the blood–brain interfaces, suggesting that LPS enters the brain under the physiological condition, possibly through a lipoprotein transport mechanism [69]. LPS injection in pig increased the expression of proinflammatory cytokine (IL-1 receptor agonist, IL-6, TNF-α, and IFN-γ) and decreased the level of noradrenaline in the hypothalamus, hippocampus, and frontal cortex compared to the control pigs, indicating the role of LPS on the changes in brain cytokines and neurotransmitter levels in pigs [70]. Another study also reported that LPS exposure induced the neuroinflammation by increasing the expression of TNF-α, COX-2, NOS-2, and IL-1β and activating the signaling of TLR4 and NF-κB in the cortex and hippocampus of adult mice compared to the control mice [71]. In addition, LPS induced neuronal degeneration by augmenting the level of Bax/Bcl2 and decreasing the activation of cytochrome-c, caspase-3 and cleaving PARP-1 in the cortex and hippocampus of a mouse brain [71]. However, TLR4 is also important for vascular endothelia and microglial activation and neuroprotection against experimental brain injury [72]. In which, they also reported that lower level of neuronal cell death and lesion volumes in mice treated with LPS and followed experimental brain injury, suggesting that a peripheral injection of LPS may induce transient CNC inflammation and neuroprotection without any apparent effects [72]. In addition, LPS induced the expression of matrix metalloproteinase-9 (MMP-9) and cell migration in astrocytes via the activation TLR4/P13K/Akt/MAPKs pathways and [73]. The mice treated with LPS induced neuroinflammation and cognitive impairment through the activation of microglial cells and NF-κB pathways [74], and induction of amyloidogenesis, memory dysfunction, and neuronal cell death [72]. Moreover, the higher levels of nitric oxide (NO), prostaglandin E2 (PE2], IL-1β, TNF-α, and Aβ_1-42_, as well as the lower levels of IL-10 and IL-4, were found in a mice model of LPS [74,75]. Krüppel-like factor 4 (KLF4) is a transcription factor that also involves in the LPS-induced microglial activation. The KLF4 knockdown decreases NF-κB activation and NO and proinflammatory cytokine productions in vitro [76]. Therefore, these studies suggest that LPS can induce neuroinflammation after migrating from the gut to the brain via circulation (Figure 3).

### 2.3. Gut Microbiota on CNS and ENS Disorders

Gut–brain crosstalk not only maintains the healthy status, but, also, in the context of gut–brain axis disruption, it has been implicated in the development of psychological and neurological diseases [77,78]. The gut bacteria have a major impact on brain development, behavior, and host immune system. However, the increased intestinal permeability causes translocation of gut microbes and their neuroactive metabolites and components that induce a neuroinflammatory response in the brain.

#### 2.3.1. Alzheimer’s Disease

Alzheimer’s disease (AD) is a common neurodegenerative disorder in which there is a deterioration in activities, memory, thinking, language, and cognitive ability that, all together, is named as dementia in older adults. The over production and deposition of amyloid-β peptide (Aβ) and translocation of microbes and their products infiltrate into the brain, where they may initiate neuroinflammation and neurodegenerative alternation in AD; therefore, the AD is often associated with increasing cerebral accumulation of Aβ [79,80]. The Aβ is a 40–42 amino acid Aβ42 peptide that derived by proteolytic cleavage of amyloid precursor protein (APP). It plays a major role in the AD pathogenesis by initiating neuroinflammatory response in AD [81]. The APP transgenic mice had a remarkable shift in the gut microbiota diversity than non-transgenic wild-type mice [82]. In addition, a drastic decrease in the level of the cerebral Aβ amyloid pathology was found in GF APP transgenic (TG) mice compared to the control mice with the gut microbiota. Intriguingly, TG mice that received gut microbiota from conventional APP transgenic mice showed an increase in cerebral Aβ pathology, while TG mice colonized with microbiota from wild-type mice had less of an effect in augmenting the cerebral Aβ level [82]. Similar results were also obtained by Bauerl et al. [83], who reported that shifts in the gut microbiota composition was observed in the transgenic APP/PS1 mice model of AD. The inflammatory related *Erysipelotrichaceae* family was found to be higher in TG mice when compared to wild-type control mice [83]. Moreover, a reduced Aβ pathology was found in transgenic APP/PS1 mice than conventional mice [84], which suggesting that the gut microbiota might play a role in Aβ pathology as well as AD pathogenesis. In another in vivo study, an increase in brain deposition of the Aβ peptide, Tau protein, COX-2, and CD11b and decrease in the level of postsynaptic density protein 95 (PSD-95) and synapsin I expression were observed in TG APPswe/PS1dE9 mice [85]. However, TG mice transplanted with fecal microbiota from WT mice improved the amyloid peptide, p-tau protein level, synaptic plasticity and alternation in gut microbiota composition as compared to WT mice [85]. In addition, an increased intestinal Aβ load, AβPP, CD68, and p-Tau immunoreactivity was observed in AD patients, as well as in APP/PS1 mice, suggesting that the intestine of AD patients may mimic the brain, and induce inflammatory and immune changes relating to AβPP and Aβ pathology [86]. Furthermore, the examination of modulatory property prebiotics against AD exhibited that fructooligosaccharides ameliorated cognitive deficits and neurodegeneration in TG APP/PS1 mice by upregulating the levels of PSD-95, synapsin I, and GLP-1 and decreasing the level of p-JNK and GLP1R [87]. Recently generated animal model of AD, an AD-like pathology with amyloid and neurofibrillary tangles (ADLP^APT^) TG mice showed amyloid plagues, neurofibrillary tangles, reactive gliosis in the brain with memory defects, and loss of intestinal barrier integrity and intestinal inflammation that were ameliorated by transplantation of the gut microbiota from wild-type mice [88]. The amyloid-β plaque formation and reactive gliosis (microgliosis and astrocytosis) are not important for the induction of cognitive deficits in APP knock-in mice (APP^NL-G-F/NL/G-F)^ model of AD [89]. They also reported that APP^NL-G-F/NL/G-F^ mice showed spatial memory deficits, while APP^NL/NL^ mice exhibited intact spatial learning and memory that were relatively similar to WT mice [89]. In addition, Reference Leblhuber et al. [90] examined the fecal concentration of calprotectin in 22 patients with AD. They found the signs of enteric inflammation with higher level of calprotectin in the AD patients.

In addition, altered gut microbial composition was observed in AD patients, suggesting that it may be involved in the AD pathogenesis [91]. Elderly patients with AD had a lower abundance of butyrate producing bacteria (*Butyrivibrio*, *Eubacterium*, *Clostridium* sp. strain SY8519, *Roseburia hominis,* and *F. prausnitzii*) and higher proportion of taxa that are associated with neurological disorders (*Odoribacter splanchnicus*) and proinflammatory state (*Bacteroides vulgatus*, *B. fragilis*, and *Eggerthella lenta*) in vivo [92]. In addition, stools samples from AD patients induced lower production of anti-inflammatory p-glycoprotein in vitro than samples from elders without AD, indicating that the association between gut microbiota and brain is the modulation of gut homeostasis by increasing inflammatory state and by decreasing anti-inflammatory response and microbial metabolisms [92]. Dysregulated gut homeostasis and changes in gut microbiota were observed in symptomatic AD mice [93]. Moreover, Vogt et al. [94], who characterized the taxonomic composition of fecal microbiota from 25 AD patients and found a decreased microbial diversity that is compositionally differed from age and sex matched subjects. Moreover, the genes wide differences were found, with decreased *Firmicutes* and *Bifidobacterium*, and increased *Bacteroidetes* in AD patients and these changes were strongly correlated with Aβ pathology and p-tau protein in the subgroup of patients [94]. Elderly patients had no definitive AD, but had decrease in the abundance of anti-inflammatory *E. rectale* and *B. fragilis* and increase in abundance of inflammatory taxon *Escherichia*/*Shigella* with higher level of IL-1β, CXCL2, NLRP3, and Aβ peptide as compared to healthy controls and subjects with cognitive impairment but had no Aβ pathology [95]. The changes of gut microbiota and associated immune responses were also showed in Table 1 [96,97,98,99,100,101,102,103,104,105,106,107,108,109,110,111,112,113,114,115,116,117]. It has also been reported that proinflammatory gut bacteria-mediated dysbiosis may induce neuroinflammation and cerebral Aβ accumulation in in AD patients, especially *Salmonella*, *Bacillus*, *Mycobacterium*, *E. coli*, and *Staphylococcus* [118]. The impact of the gut microbiome on AD pathogenesis is not limited to bacteria, but viruses have also been reported to nexus with AD [119]. However, still more studies are needed to better understanding the role of the microbiota on the gut–brain axis in AD.

#### 2.3.2. Autism Spectrum Disorder

Autism spectrum disorder (ASD) is another important neurological disorder, which defines a group of neurodevelopmental disorders characterized by impaired social communications and interactions in addition to repetitive and restrictive patterns of behavior with disturbed anxiety and cognitivist. There is a growing evidence supporting the role of gut and the resident microbiota on the severity of ASD. The hypothesis of a strong correlation between the disruption of gut bacteria and ASD is mainly ordinated from the clinical studies that children with ASD appear to have gastrointestinal problems/symptoms, such as constipation and diarrhea. Intriguingly, ASD children treated with antibiotic vancomycin ameliorated the severity of ASD and improved the behavioral symptoms, suggesting that the gut bacteria may participate in the behavioral disturbances in ASD [120,121]. Several other studies have also found remarkable changes in the gut microbiota composition and in the production of metabolites in children with ASD [107,122]. The relative abundance of *Lactobacillaceae*, *Bifidobacteraceae*, and *Veillonellaceae* were found to be higher in ASD children, while healthy children had higher proportion of *Prevotellaceae* [105]. In another study, children with ASD had relative decrease in the abundance of *Acidaminococcaceae*, *Lachnoclostridium*, *Flavonifractor,* and unidentified *Lachnospiraceae* than healthy controls [106]. The composition of SCFAs was also altered in ASD subjects, with a decrease in the level of fecal acetic acid and butyrate, and an increase in the level of fecal valeric acid were observed in ASD subjects [107]. Moreover, the lower level of butyrate-producing taxa of gut microbiota (*Ruminococcaceae*, *Eubacterium*, and *Lachnospiraceae*) and higher level of valeric acid associated bacteria (*Acidobacteria)* were found in ASD subjects, suggesting that gut microbiota contribute to ASD pathogenesis [107] (Table 1). Children with ASD also had significantly higher level of fecal isopropanol and p-cresol and lower level of GABA concentration [108]. The authors found that the gut microbial diversity and the relative abundance of *Prevotella copri*, *F. prausnitzii,* and *H. parainfluenza* were decreased in the feces of children with ASD [108]. In the small open-label clinical study, the microbiota transfer therapy (MTT) in children with ASD, was significantly improved the gastrointestinal and ASD symptoms in children [121,122]. The bacterial diversity and the abundance of *Prevotella*, *Bifidobacterium*, and *Desulfovibrio* taxa were found to be higher in ASD children with MTT treatment. The gastrointestinal disturbances and the alternation in the composition of the gut microbiota were restored or reversed by probiotics [123,124] and dietary [125] treatment in ASD children.

The phylum *Bacteroidetes* is a known producer of SCFA that was found to be higher in ASD patients. In addition, the lower level of anti-inflammatory *Bifidobacterium* and a higher level of phenol and p-cresol producer *Clostridium* were observed in ASD patients [109]. Along with this, the changes of gut microbiota increase the production of intestinal serotonin and reduce the production of cerebral serotonin in subjects with ASD [109]. In addition, changes in the gut microbiota composition and its metabolites have been observed in animal with ASD [126]. In a mouse model, the composition of gut bacteria in animal with ASD was differed from control animal. However, this alternation in the gut bacterial composition was restored when the mice treated with *B. fragilis*, and remarkably ameliorated the stereotyped and anxiety-like behavior, indicating the nexus of the gut–brain axis on the modulation of neurodevelopmental disorders [127].

#### 2.3.3. Parkinson’s Disease

Parkinson’s diseases (PD) is the second most common neurodegenerative disorder and estimated about seven to ten million people suffering from PD worldwide [128]. PD is mainly characterized by both motor and non-motor disturbances. On the motor side, slowness of movement, rigidity and resting tremor are the most prominent symptoms. On the non-motor side, cognitive disturbances, depression, mood deflection, sensory alternations, sleep alternations, autonomic dysfunctions contribute to significant disability. Such widespread clinical spectrum reflects the accumulation of α-synuclein in both central and peripheral nervous system [129,130]. Gastrointestinal (GI) symptoms are experienced by most PD patients. Various GI symptoms such as hypersalivation, dysphagia, constipation, nausea, altered bowel habits, and defecatory dysfunction were reported to present in patients with PD [131]. Increased intestinal permeability has been observed in the early stages of PD [132]. Higher intestinal permeability induces translocation of gut bacteria and microbial components that initiate inflammation and oxidative stress in the ENS, resulting in enteric α-synucleinopathy in PD [132]. Since the 1960s, *Helicobacter pylori* (HP) infection and the related complication (gastric ulcers) have been reported to be associated with PD [133]. The eradication of HP infection by using drugs ameliorates PD symptoms [134]. The supporting evidence from the several studies hypothesizes a relationship between the gut and the resident microbiota and PD. Gut bacterial alternations and inflammatory state are the important co-factors involved in PD. Fecal sample analysis showed that the significant alternation in the gut bacterial composition was observed with lower level of *Prevotellaceae*, and higher level of *Enterobacteriaceae* in PD patient cohort. These changes were positively correlated with higher level of postural instability and distinctive gait [101]. GF mice received fecal microbiota from PD patients showed an increase in the level of α-synuclein, a presynaptic neuronal protein that is associated with motor dysfunction and neuroinflammatory state in mice [135]. A study demonstrated in animal was also found the changes in the gut microbiota composition in chemical induced PD mice model [98]. A recent study showed that the relative abundance of mucin-degrading *Verrucomicrobiae* and LPS producing *Gammaproteobacteria* were found to be higher in both PD patients and a human α-synuclein over expressing mice model of PD (Thy-αSyn), as compared healthy and wild-type controls. LPS administration increased the early motor manifestation in Thy-αSyn mice at 10 weeks [97]. The neuroprotective effect of gut microbiota on MPTP-induced mice model of PD was demonstrated by Reference [100]. They found that fecal microbiota transplantation (FMT) decreased microbial dysbiosis, fecal SCFAs, and physical impairment and elevated striatal DA and 5-HT in a mouse model of PD. They also reported that FMT protects PD mice by reducing the activation of microglia and astrocytes, as well as by reducing the signaling of TLR4/TNF-α in gut and brain [100]. A recent study reported the increased colonic expression of TLR4, CD3^+^T cells, and cytokines and decreased abundance of SCFAs producing bacteria in PD patients [103]. Authors were also demonstrated in TLR4-KO mice, in which they found the similar gut and neurological disorders that could be reversed/reduced by rotenone treatment, suggesting that the TLR4-induced inflammatory signaling plays vital role in gut and brain inflammation in PD [103]. The increase in the level of microglial activation, proinflammatory cytokine production, and heme oxygenase-1 (HO-1), and the decrease in the level of ferroportin (Fpn) were observed in the LPS-induced PD mice models [99].

#### 2.3.4. Multiple Sclerosis

Multiple sclerosis (MS) is also a prevalent CNS-related neurological disease that elicits autoimmune disease on the myelin sheath. It is characterized by blurred vision, motor dysfunction, and changes in sensibility [136]. There is evidence of alternation in the gut microbial composition in MS [137]. Several studies reported that the profile of gut microbiota in MS patients was different from healthy individuals [112,137]. The gut microbial changes increase the level of regulatory T cells (Treg) that reduce the activation of proinflammatory T cells [138]. The elevated levels of circulatory Th1 and Th17 cells increased the blood–brain barrier permeability (BBB) and induced inflammation in CNS [136]. Considerably, the transplantation of fecal microbiota from MS patients into transgenic mice induced a higher incidence of autoimmune encephalomyelitis (EAE) than mice transplanted with the microbiota from healthy individuals [138]. Mice treated with a broad spectrum of antibiotics prevented motor dysfunction and axon dame in a mice model EAE, while bacterial recolonization impaired the motor function and axon integrity [139]. The protective mechanism of antibiotics is probably through the involvement of CD4^+^CD39^+^ T cells ad CD5^+^CD1d^+^ B cells in the CNS of mice [139]. Similarly, the antibiotics treated mice delayed the onset of clinical of symptoms of EAE and decreased the level of IFN-γ and IL-17A and increased the level of IL-10 in serum of EAE mice [140]. They also found that the depletion of microbiota by antibiotics could decrease hippocampal BDNF and increase learning and memory levels in EAE induced mice. In addition, they reported increases in anxiety-like behavior and hippocampal TNF-a and IL-1β, as well as decreases in depression-related symptoms, in EAE-induced mice [140]. These studies confirmed the association of the gut microbiota on the neurodegenerative disease severity in the model of progressive MS.

#### 2.3.5. Amyotrophic Lateral Sclerosis

Amyotrophic lateral sclerosis (ALS) is another fatal neurodegenerative disease that is characterized by muscle weakness and the progressive loss of upper and lower motor neurons in the brain, brainstem, and spinal cord [141]. ALS also causes cell death and neuroinflammation in the brain and spinal cord. The abnormalities such as mitochondrial dysfunction, glutamate excitotoxicity, changes in the RNA metabolisms, microglial and astrocyte activations, and autophagy dysregulation were observed with ALS [142]. However, ALS etiology and pathophysiology are not well explored. Recent studies suggest that the gut dysbiosis and altered gut bacterial composition contribute role in ALS etiology and progression of ALS [143,144]. Recently, a prospective longitudinal study characterized the microbiota composition in ALS patients [145]. An unbalance between protective microbial groups (*Bacteroidetes*) and neurotoxic/proinflammatory groups (*Cyanobacteria*) was observed in patients with ALS. The genera belonging to *Enterobacteriaceae*, *Akkermansia*, *Eubacterium*, *Prevotellaceae* and *Ruminococcaceae* were found to be higher in ALS patients. In contrary, genera belonging to *Veillonellaceae* and *Lachnospiraceae* families, the genus *Parasutterella*, *Ruminococcus* and *Subdogranulum* were more abundant in control group [145]. In addition, differences in the microbial biodiversity between ALS patients and controls were observed. The Chao1 index (alpha-diversity), associated to the abundance of sequences for each operational taxonomic units (OUT) was significantly higher in the control group than ALS group [145]. The analysis of fecal microbiota showed significant changes in the microbial composition of ALS patients, where *Bacteroidetes* at phylum level and *Kineothrix*, *Parabcateroides*, *Odoribacter*, *Sporobacter*, *Eisenbergiella*, *Mannheimia*, *Anaerotruncus*, and unclassified *Porphyromonadaceae* at genes levels, were found to be higher compared to control group [146]. In contrast, a significant reduction in *Firmicutes* at phylum level and at *Megamonas* at the gene level was observed in the ALS group, in comparison to the control group. Furthermore, pathways relating to metabolism of amino acids, nucleotide, and carbohydrate were found to be lower in ALS group than control group [146]. A comparative study was also evaluated bacterial and archaeal composition of gut microbiota and metabolism in ALS patients [147]. There was significant difference observed in the bacterial composition between ALS patients and healthy individuals. The phylum level *Firmicutes/Bacteroidetes* ratios was relatively higher in patients with ALS, whereas the beneficial bacteria, such as *Faecalibacterium* and *Bacteroides,* at the gene levels, were lower in ALS patients, as compared to healthy individuals. In addition, the endotoxin, NO2-N/NO3-N, and gamma-aminobutyric acid were found to be higher in patients with ALS than healthy controls [147]. ALS-prone *Sod1* transgenic (*Sod1*-Tg) mice had dysbiosis and altered metabolite configuration. Antibiotic treatment worsens the disease severity under GF conditions [148]. The commensal *Akkermansia muciniphila* supplementation ameliorates ALS symptoms, while *Ruminococcus torques* and *Parabacteroides distasonis* exacerbate the symptoms of ALS in Tg Mice [148]. These studies suggest that gut microbiome may have an etiological role in ALS.

#### 2.3.6. Huntington’s Disease

Huntington’s disease (HD) is a progressive neurodegenerative disorder that is characterized by a triad of clinical features, namely progressive motor, cognitive, and psychiatric impairments, as well as unintended weight loss [149]. It is mainly caused by the expansion and unstable trinucleotide (cytosine-adenine-guanine, CAG) repeat in the huntingtin (HTT) gene that is expressed in through the brain [150]. In addition, neuronal degeneration in the basal ganglia, white matter atrophy, and myelination deficits represent early pathological features of the HD in both patients and animals [151,152]. Recently, some studies have pointed to the possible gut dysbiosis in HD. The unintended weight loss is a characteristic clinical feature of HD and has been induced by gastrointestinal dysfunction in an HD mice model [153]. A study in animal model reported that a significant difference in microbial composition was noted in HD mice at 12 weeks of age. Specifically, an increase in level of *Bacteroidetes* and decrease in level of *Firmicutes* were observed in HD mice [154]. In addition, gut dysbiosis and motor deficits were observed at 12 weeks of age in HD mice. GF condition altered callosal myelination and white matter plasticity in a bacterial artificial chromosome model of HD mice [155]. Wasser and coworkers analyzed the gut microbiota composition in people with HD. The authors found significant differences in the gut microbial communities (beta diversity), and lower alpha-diversity (richness and evenness) between combined HD gene expansion carrier group and healthy controls [149]. The *Euryarchaeota*, *Firmicutes,* and *Verrucomicrobia* at phylum level were significantly differed between male groups. At the family level, *Acidaminococcaceae*, *Akkermansiaceae*, *Bacteroidaceae, Bifidobacteraceae*, *Clostridiaceae*, *Christensenellaceae*, *Coriobacteriaceae*, *Eggerthellaceae*, *Enterobacteriaceae*, *Erysipelotrichaceae*, *Flavobacteriaceae*, *Lachnospiraceae*, *Methanobacteriaceae*, *Peptococcaceae*, *Peptostreptococcaceae*, and *Rikenellaceae* were also significantly differed between male groups [149]. In addition, the associations among gut microbiota, cognitive performance, and clinical outcomes were discovered within the HD gene expansion carrier group.

#### 2.3.7. Gut Microbiota on Depression

Depression is a common disorder, but the most serious mental illness worldwide is now known as major depressive disorder (MDD). It has been reported that MDD is associated with an increase in the level of proinflammatory cytokines and alternation of gut microbiota composition in both human [105] and animal depressive model [156,157]. An increase in the level of *Bacteroidetes* and *Proteobacteria* and decrease in the level of *Firmicutes* were observed in depressive patients [105]. Recently, in a large microbiome population cohort study, the *Dialister* and *Coprococcus* spp. were depleted in people with depression [158]. The authors also reported that the gut microbiota were capable of synthesizing neurotransmitter dopamine (3,4-dihydroxyphenylacetic acid, DOPAC) in depressive persons [158]. Patients with depression who committed suicide had significantly smaller urinary outputs of DOPAC and homovanillic acid and total body outputs of sum dopamine than patients with depression who did not commit suicide and normal control subjects [114]. Recently, a group investigated the relationship between gut microbiota and MDD, using a PubMed literature search [117]. The authors reported that a higher abundance of nine genera (*Anaerostipes*, *Blautia*, *Clostridium*, *Klebsiella*, *Lachnospiraceae incertae sedis*, *Parabacteroides*, *Parasutterella*, *Phascolarcobacterium,* and *Streptococcus*) and lower level of six genera (*Bifidobacterium*, *Dialister*, *Escherichia/Shigella*, *Faecalibacterium*, and *Ruminococcus*) were found in MDD [117]. Much of what is known is mainly from the earlier studies that showed the link between the gut microbiota and depressive disorder; however, the mechanisms by which microbes shape the CNS functions, and how they play a role in mental illness, are still controversial.

Overall, gut microbiota have the potential to improve brain function, as well as the progression of neurological disorders.

### 2.4. Therapeutic Treatment

#### 2.4.1. Probiotics/Prebiotics/Synbiotics/Antibiotics

Nowadays, the pro/pre/synbiotics are increasingly being used in several fields, including the medical and clinical fields. The common term of probiotics is “live microorganisms that which confers a health benefit on the host when ingested in adequate amounts”. Prebiotics are non-digestible food fibers that beneficially affect the host health by selectively increasing the growth and activity of gut microbes, especially *Lactobacillus* and *Bifidobacterium* [159]. Synbiotics means a combination of both prebiotics and probiotics. The most common beneficial effects of probiotics are restoring the gut microbiota, improving intestinal and immune homeostasis [160,161]. In addition, probiotics were reported to exhibit modulatory effects on CNS disorders, including normalization of anxiety and depression-like behaviors [35,162] and reduction of ASD [163]. A randomized, double-blind, placebo-controlled study reported that the consumption of probiotic *L. plantarum* PS128 for four weeks significantly improved ASD associated symptoms, comparted with the placebo group [164]. The probiotic treatment was also significantly improved the brain function in mice with AD model [165]. Razaeiasi et al. [166]. have analyzed the effects of probiotics on spatial learning and memory and some other parameters in rats with AD. Rats that received probiotics (*L. acidophilus*, *B. bifidum,* and *B. longum*) for four weeks showed significant improvement on spatial learning and memory, long-term potentiation (LTP), paired-pulse facilitation (PPF) ratios, and lipid profiles, as compared to the AD group. In addition, the oral administration of probiotics (*L. reuteri*, *L. rhamnosus,* and *B. infantis*) significantly improved spatial memory and decreased Aβ plaques, oxidative (MDA), and inflammatory (IL-1β and TNF-α) markers in rats at 10 weeks [167]. The probiotic (*L. paracasei*, *L. plantarum*, *L. acidophilus*, *L. delbrueckii*, *B. longum*, *B. infantis*, *B. breve,* and *Streptococcus thermophilus*) administration daily, for two months, improved MS symptoms by modulation of gut microbiota and anti-inflammatory peripheral immune response in MS patients [168]. In addition, probiotic *S. thermophilus* ST285 reduced proinflammatory (IL-1β and IFN-γ) and anti-inflammatory cytokines (IL-4, IL-5, and IL-10) in mice immunized with multiple sclerosis peptide [169]. The probiotics supplementation for six months influenced gut bacterial composition, especially *Rikenellaceae* at family in ALS patients compared to compare to control group [145]. The probiotic formulation (SLAB51) has been reported to attenuate the cognitive impairment, Aβ aggregation, brain injuries, and alternation of neuronal proteolysis in AD mice [170]. It also promotes antioxidant and neuroprotective effects via the activation of SIRT1 pathway in AD mice model [171]. Administration of probiotics (*L. acidophilus*, *B. bifidum,* and *B. longum*), along with selenium, for 12 weeks, improved cognitive functions and some metabolic parameters in AD patients [172]. Probiotics plus selenium intake resulted in significant reduction in high sensitivity CRP, insulin, serum triglycerides, HOMA-IR, VLDL, and LDL levels and significant increase in total antioxidant capacity compared with only selenium and placebo [172]. In a randomized, double-blind, placebo-controlled study, 12 weeks of *B. breve* A1 supplementation improved cognitive function in elderly subjects with memory complaints [173]. In an open-label single-arm study, intake of the same strain *B. breve* A1 was significantly improved hospital anxiety and depression scale (HADS) score and Positive and Negative Syndrome Scale (PANSS) at four weeks [174]. Additionally, the probiotic intake significantly increased the levels of IL-22 and TRANCE expression at weeks [174]. A meta-analysis of randomized controlled trials showed that probiotics intake improved cognitive functions in AD and mild cognitive impairment (MCI) subjects, possibly via decreasing the levels of inflammatory and oxidative markers [175]. In contrast, the meta-analysis of randomized controlled trials showed no remarkable difference between probiotics/prebiotics and placebo in alleviating anxiety and depressive symptoms [176,177]. Recently, in vitro, probiotics formulation (SLAB51) exhibited neuroprotective effect by modulating the brain derived neurotrophic factor (BDNF) pathway, increasing P13K/Akt, pTrK, pERK5, and p-CREB pathways, and decreasing p-JNK, ERK-1, and P75 pathways in human neuroplastoma cells [178]. In addition, the probiotic administration has been shown to improve behavioral impairments and protect dopaminergic neurons of *substantia nigra* and *striatum* in PD mice [178]. Probiotics *L. salivarius* (LS_01_) and *L. acidophilus* (LA_02_) were able to significantly decrease the levels of proinflammatory cytokines and reactive oxygen species (ROS) and increase the anti-inflammatory cytokines in Peripheral blood mononuclear cells (PBMCs) from PD patients and healthy controls [179]. In a randomized, double-blind, placebo-controlled study, the consumption of fermented milk with multiple probiotic strains and prebiotic fibers has been shown to improve constipation in PD patients [180]. PD patients receiving fermented milk containing *L. casei Shirota* improved stool consistency and decreased bloating and abdominal pain [181]. In addition, the administration of capsule form of probiotics (*L. acidophilus*, *L. reuteri*, *L. fermentum*, and *B. bifidum*) for 12 weeks, resulted in useful impacts on Movement Disorders Society Unified Parkinson’s Disease Rating Scale (MDS-UPDRS) and some metabolic profiles in PD patients [182]. Probiotic consumption has also reduced the levels of high-sensitivity C-reactive protein, malondialdehyde, insulin, and insulin resistance and increased glutathione and insulin sensitivity, in comparison with the placebo [182].

**Figure 4:**
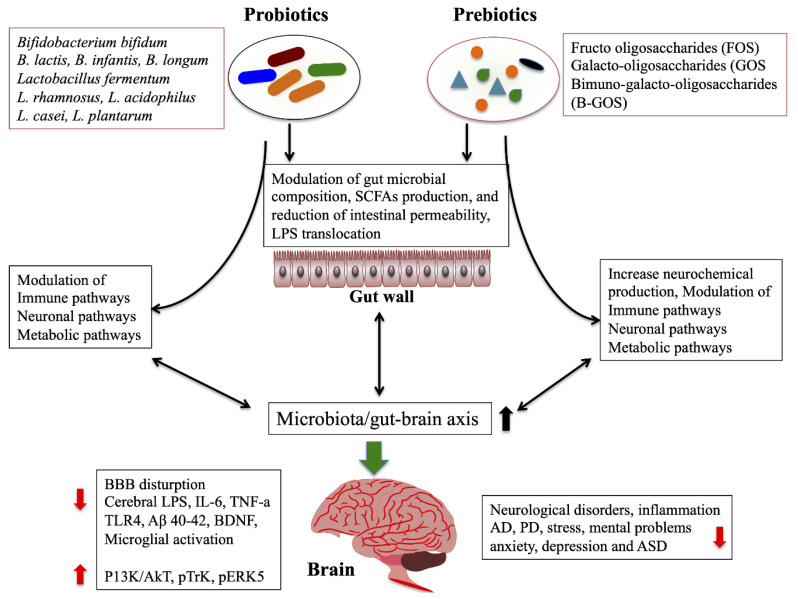
Effect of probiotics/prebiotics on improvement of microbiota/gut–brain axis. The presence of probiotics and prebiotics or combination of both was able to improve neurological complications by increasing the production of SCFAs and neurochemicals, reducing the intestinal permeability, and modulating the gut microbial composition, immune, metabolic, and neural pathways. (↑ ↓ indicate up and down-regulation).

Probiotic-4 (*B. lactis*, *B. bifidum*, *L. casei*, and *L. acidophilus*) administration was able to attenuate the aging-related disruption of blood–brain barrier and intestinal barrier integrity and reduce the level of plasma and cerebral LPS, as well as IL-6, TNF-α and TLR4, and NF-κB translocation in the brain of aged mice [183]. In addition, the remarkable improvement of memory deficits, cerebral neuronal and synaptic injuries, microglia activation, and microbial composition were found in the feces and brain of aged mice model [183]. Chronically stressed mice treated with *B. breve* CCFM1025 reversed chronic stress-induced depression and anxiety-like behaviors and gut microbiome changes in vivo [184]. It has increased the expression of BDNF, SCFAs, and 5-hydroxytryptophan (5-HTP) and decreased the pCREB-c-Fos pathway. *Akkermansia muciniphila* (Akk) is a type of gut bacteria that exhibits probiotic effects against several diseases. Akk fed APP/PS1 mice showed reduced the levels of fasting blood glucose, lipids and serum diamine oxidase, as well as the level of cerebral Aβ 40–42 in vivo [185]. Prebiotics have also been reported to improve brain function and prevent neurological disorders AD [186], dementia [187], IBS [188], PD [189], and ASD [190]. The fructo (FOS) and galacto-oligosaccharides (GOS) treatments exhibit beneficial effects on chronic stress mice by improving the depression and anxiety like behaviors via targeting the microbiota-gut–brain axis [191]. The administration of prebiotics was able to increase cecal SCFAs (acetate and propionate) and to reduce chronic stress induced proinflammatory cytokines and corticosterons in stress-induced mice. Similarly, the prebiotic Bimuno-galacto-oligosaccharides (B-GOS) treatment alleviated cognitive functions and significantly dampened microglia activation as well as the expression of iNOS, CD68, CD32, SOCS3, and IL-6 in rat [192]. In addition, it significantly increased beta diversity of the gut microbiome and *Bifidobacterium* proliferation and other anti-inflammatory microbes in rat, suggesting that prebiotics had potential effect on brain function through the gut–brain axis. A pilot study showed that the administration of synbiotics improved cognitive functions in patients with hepatic encephalopathy [193]. Human milk oligosaccharides (HMOS) are also the infant prebiotics that showed symbiotic effects along with *B. longum* subsp. *infantis* in an in vitro and humanized mice model [194]. Combination of both reduced the levels of *Clostridia* and G-bacteria, while increased the production of IL-10 and IL-6 in both in vitro and in vivo mice model. A novel synbiotic (Triphala and *L. plantarum, L. fermentum*, and *B. longum* subsp. *infantis*) increased the motility and survivability, while reducing the deposition of Aβ and acetylcholinesterase activity in transgenic humanized Drosophila model of AD [195]. It also reduced the inflammatory, immune and oxidative markers and mitochondrial stress via implicating PPAR-γ pathway. This mood illness has been improved when patients were treated with synbiotics [196], probiotics of multiple strains (*L. acidophilus*, *L. casei* and *B. bifidum*) [197], *L. rhamnosus* HN001 [198], and *C. butyricum* [199], and probiotic mixture [200]. In addition, supplements such as polyunsaturated fatty acids (PUFAS), especially eicosapentaenoic acid (EPA), and folate-based N-acetlycysteine are the strongest adjunctive treatment for depressive disorder [201]. A systemic review reported that probiotics, prebiotics, and synbiotics are very useful therapeutic methods for the improvement of cognitive functions, behavioral and psychological symptoms in patients with dementia [187]. Over all, how probiotics/prebiotics improve brain function via regulation of microbiota/gut–brain axis was simplified by diagram (Figure 4).

As said earlier, gut microbiota play a vital role in neurological disease including AD. The administration of antibiotics has been shown to alter the gut bacterial composition, which positively or negatively affects the brain functions. Several studies showed negative results with increasing serum cognitive and anxiety-like behaviors in vivo [202,203]. However, these adverse negative effects of depend on the type and functions of antibiotics. The antibiotics omeprazole, amoxicillin, and clarithromycin reduced the *Helicobacter pylori* counts that positively affect the brain functions in patients with AD [204]. The non-absorbable antibiotics treatment counteracts the 6-hydroxydopamine (6-OHDA) induced dopaminergic neuronal loss, proinflammatory cytokines production in the striatum, and the degree of motor dysfunction in the PD rat model [205]. The rifampicin, rapamycin, and minocycline administration reduced the level of Aβ, inflammatory cytokines, and microglial action in AD mice [206,207,208].

#### 2.4.2. Fecal Microbiota Transplantation

Fecal microbiota transplantation (FMT) is a potent treatment method for several diseases. Recently, it has been increasingly used for the treatment of several neurological diseases. The open-label clinical studies showed that FMT improved the gastrointestinal and ASD symptoms in children with ASD [123,209]. The FMT from inflammasome NLRP3 KO mice were significantly ameliorated the depressive-like behavior induced by chronic unpredictable stress (CUS) in mice [210]. The expression level of circular RNA HIPK2 (circHIPK2) was found to be higher in the mice treated with CUS than the control. In addition, the FMT significantly reduced astrocyte dysfunction in CUS-treated mice through the inhibition of circHIPK2 [210]. Another recent study also examined the FMT and its impacts on depressive disorder in mice. Mice transplanted with fecal microbiota from chronic unpredictable mild stress (CUMS) mice increased anxiety-like and depression-like behavior compared to the control [211]. In addition, there were higher levels of IFN-γ, TNF-α and indoleamine 2,3 dioxygenase 1(IDO1) in the hippocampus of the recipient mice, indicating that gut microbiota modulate inflammatory response in the hippocampus via disruption of the microbiota-gut–brain axis to exacerbate anxiety and depressive-like symptoms [211]. MS patients received FMT showed improvement in disease progression [212] and MS symptoms [213]. Fecal microbiota from AD patients to GF mice affect mouse behaviors and less production of γ-aminobutyrate, taurine, and valine in vivo [214]. The 71-year-old PD patients had FMT from healthy young donors. The patients’ tremor in the legs and gastrointestinal symptoms were improved after FMT [215].

In summary, several neurological insights into the gut–brain axis reveal that the gut microbiota have strong bidirectional communication with the CNS and control the development and functions of the CNS that, in turn, improves the gut homeostasis. Abnormalities in the brain functions affect the GI physiology, including digestion and gut microbial composition. The mechanisms behind this axis are very complex and several pathways involves directly and indirectly. Intestinal permeability is the promising aspects of influencing or affecting the CNS/ENS functions. Gut-derived metabolites and components are the key factors that translocate to the brain and disturb BBB and microglial activation and induce inflammatory immune pathways. However, mechanisms that directly affecting the CNS/ENS functions via gut-derived metabolites/components remains unclear. Testing of therapeutic strategies, such as probiotics, prebiotic, dietary components, and FMT in patients with neurological problems and altered gut microbiota composition would be more valuable and increase our understating behind the beneficial or pathological role of the gut microbiota on the brain through the gut–brain axis.

## Figures and Tables

**Table 1:** Alternation of gut microbiota and related immune/inflammatory effects in animal and patients with neurological disorders.

Neurological Disorder	Models	Changes of Microbiota/Composition	Immune/Inflammatory Effects	References
AD	APP/PS1 and Tg2576 animals	*Firmicutes, Verrucomicrobiae, Proteobacteria, Actinobacteria,* *Rikenellaceae* and GS247, *Turicibacteriaceae, Erysipelotrichaceae,* and *Allobaculum* and *Akkermansia Lactobacillus* and *Ruminiclostridium*	Increase in cerebral Aβ pathology and IL-1β production. Plasma MCP-1 was high in symptomatic mice, while IL-9, VEGF-α, and IP-10 were higher in pre-symptomatic Tg2576 mice.	[82,83,93]
	Human Patients	*Odoribactersplanchnicus, Bacteroides vulgatus, B. fragilis, Eggerthella lenta, Escherichia/Shigella, Gemella, Butyrivibrio, Eubacterium, Clostridium, Roseburia hominis, Bifidobacterium, F. prausnitzii*	Increase in cerebral Aβ accumulation and neuroinflammation. Bacterial LPS was found in the brain. The higher levels of IL-1β, NLRP3, and CXCL2 were positively correlated with *Escherichia/Shigella* abundance.	[92,94,95,96]
PD	Mice	*Verrucomicrobiae, Gammaproteobacteria, Erysipelotrichaceae, Akkermansia, Proteobacteria* and *Lachnospiraceae, Clostridiates*	Microglial activation, inflammatory cytokines, and HO-1 were found to be higher in PD mice. Gut inflammation; disrupted intestinal barrier with higher level of IL-17, TNF-α, and IL-1β; and activation of microglia, astrocytes, and higher level of TLR4 expression were also found in the brain of PD mice.	[97,98,99,100]
	Patients	*Enterobacteriaceae, Proteobacteria, Verrucomicrobiaceae, Lactobacillaceae Porphyromonas, Parabacteroides Prevotellaceae, F. prausnitzii, Bacteroides fragilis, B. dorei, B. pebeus, Ruminococcus callidus*	TLR4, CD3+T cells, cytokines were found to be higher in the intestine of PD patients. Higher levels of IFN-γ, TNF-α were also found in PD patients.	[4,101,102,103,104]
ASD	Patients (Childrens)	*Lactobacillaceae, Bifidobacteraceae, Veillonellaceae, Acidobacteria, Clostridium Acidaminococcaceae, Lachnoclostridium, Flavonifractor, Lacnospiracease, Eubacterium, Rumnicoccaceae, Prevotella copri, F. prausnitzii, H. parainfluenza*	Lower level of fecal acetic acid and butyrate, and higher level of valeric acid were found in ASD subjects. Increase in the level of intestinal serotonin and decrease in level of cerebral serotonin were also found in ASD subjects.	[105,106,107,108,109]
MS	Patients	*Desulfovibrionaceae, Haemophilus Verrucomicrobia, Blautia, Dorea, Pseudomonas, Mycoplana, Acinetobacter Enterobacteriaceae, Bacteroidetes, Ruminococcaceae, Heliobcateraceae, Sutterlla Lachnospiraceae, Collinsella*	Serum level of Lipid 654 was lower in MS patients. The immune markers such as Th2, Th17, and Treg did not significantly differed between both controls and MS patients.	[110,111,112,113]
Depression	Patients	*Anaerostipes, Blautia, Clostridium, Klebsiella, Lachnospiraceae incertae sedis, Parabacteroides, Parasutterella, Phascolarcobacterium and Streptococcus, Bifidobacterium, Dialister, Escherichia/Shigella, Faecalibacterium, Ruminococcus*	The lower level of neurotransmitter dopamine (DOPAC) and homovanillic acid were found in the depressive patients. In addition, decreased hippocampus 5-HT, BDNF expression and circulatory IL-10, and increased plasma stress hormone were found with depression.	[114,115,116,117]
